# Open resections for congenital lung malformations

**DOI:** 10.4103/0971-9261.43812

**Published:** 2008

**Authors:** Dhanya Mullassery, Matthew O. Jones

**Affiliations:** Department of Pediatric Surgery, Royal Liverpool Children‘s Hospital, Alder Hey, Liverpool, UK L12 2AP

**Keywords:** Pediatric lung resections, thoracotomy

## Abstract

**Aim:**

Pediatric lung resection is a relatively uncommon procedure that is usually performed for congenital lesions. In recent years, thoracoscopic resection has become increasingly popular, particularly for small peripheral lesions. The aim of this study was to review our experience with traditional open lung resection in order to evaluate the existing “gold standard.”

**Materials and Methods:**

We carried out a retrospective analysis of all children having lung resection for congenital lesions at our institution between 1997 and 2004. Data were collected from analysis of case notes, operative records and clinical consultation. The mean follow-up was 37.95 months. The data were analyzed using SPSS.

**Results:**

Forty-one children (13 F/28 M) underwent major lung resections during the study period. Their median age was 4.66 months (1 day–9 years). The resected lesions included 21 congenital cystic adenomatoid malformations, 14 congenital lobar emphysema, four sequestrations and one bronchogenic cyst. Fifty percent of the lesions were diagnosed antenatally. Twenty-six patients had a complete lobectomy while 15 patients had parenchymal sparing resection of the lesion alone. Mean postoperative stay was 5.7 days. There have been no complications in any of the patients. All patients are currently alive, asymptomatic and well. None of the patients have any significant chest deformity.

**Conclusions:**

We conclude that open lung resection enables parenchymal sparing surgery, is versatile, has few complications and produces very good long-term results. It remains the “gold standard” against which minimally invasive techniques may be judged.

## INTRODUCTION

Pediatric lung resection is most commonly performed for congenital malformations, which include congenital cystic adenomatoid malformations (CCAMs), congenital lobar emphysema (CLE), sequestrations and bronchogenic cysts. The rate of resection has increased in recent years owing to improvements in antenatal detection, with the result that many asymptomatic lesions are being removed on the basis that they pose a future risk of infection or malignancy.[1] Traditionally, open resection and /or lobectomy has been the procedure of choice; however, a number of surgeons are now advocating the use of minimally invasive techniques on the grounds that this may lead to an improved outcome.[2] The purpose of this study was to review our experience with open resection in order to establish whether there might be a significant scope for improvement.

## MATERIALS AND METHODS

This was a retrospective review. All patients with congenital lung lesions who had open resection by a single surgeon at our institution between August 1997 and November 2004 were included in the study. Patients undergoing thoracotomy for infective or malignant lesions were excluded. Data were collected from a review of case notes, operative records and patient examination. The data were analyzed using SPSS (SPSS version 12.0.1 for Windows, SPSS Inc., Chicago, IL, USA).

## RESULTS

Forty-one patients (28 M/13 F) with a mean age of 4.7 months were included in the study. The final patient who is not on the list was a 16 year old who presented with a lung abscess. The histology of his lobectomy showed evidence of CCAM. This patient was excluded from the final analysis as this is atypical, and because we do not have definite evidence to suggest that the CCAM was the pathology behind his lung abscess at such a late age.

**Figure 1:**
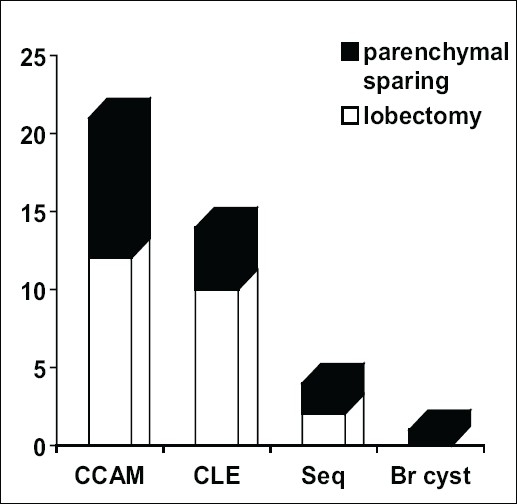
Spectrum of malformations

The lung lesions included 21 CCAMs (51.2%), 14 CLEs (34.1%), four sequestrations (9.8%) and one bronchogenic cyst (2.4%). Twenty-one (51.2%) of these lesions were diagnosed antenatally and 28 (70%) were symptomatic. Twenty-six (63%) lesions were removed by lobectomy and 15 (37%) by parenchymal sparing resection of the lesion alone [Figure 1].

There were no operative or perioperative complications. Blood loss was minimal and no patient required transfusion. Most infants were extubated at the end of the procedure and the mean ITU/HDU stay was 1.7 days (range 0–12 days). The mean inpatient stay was 5.7 days (range 3–13). These results are skewed by a single infant with a giant CCAM that was causing gross mediastinal shift, hydrops and extreme respiratory difficulties. An initial debulking procedure was performed to help ventilation, followed 3 days later by a definitive resection. Details of the patients in the major diagnostic groups are summarized in Tables 1 (CCAM), 2 (CLE) and 3 (intralobar sequestration).

**Table 1:** Details of patients with histologically confirmed congenital cystic adenomatoid malformation

No.	Age at surgery	Site	Preop symptom	AN diagnosis	PN diagnosis	Postop ITU days	Complications	FU (months)
1	6w	LLL	None	CCAM	CCAM	5	Pneumothorax + pleural effusion	48
2	16w	RML	cough + dyspnoea with feeds	CCAM	CCAM	1	None	32
3	11w	LLL	None	CCAM	CCAM	0	None	19
4	1d	Lingula	Respiratory distress after birth	CCAM	CCAM	0	None	19
5	3d	RLL	Respiratory distress after birth	CCAM	CCAM	0	None	77
6	42w	LUL	Recurrent respiratory infections	None	CCAM	1	None	57
7	12w	LUL + lingual	Cough	CCAM	CCAM	1	None	42
8	18w	RLL	None	CCAM	CCAM	1	None	35
9	60w	RLL	None	CCAM	CCAM	1	None	23
10	60w	RML	Recurrent respiratory infections	CCAM	CCAM	1	None	54
11	26w	RUL	None	Cystic lesion	CCAM	2	None	67
12	112w	LLL	Respiratory infection	CCAM	CCAM	1	None	26
13	100w	RLL	Respiratory infection	None	CCAM	2	None	43
14	26w	RML	None	CCAM	CCAM	1	Consolidation in residual middle lobe	50
15	2d	?RLL	Respiratory distress	CCAM	CCAM	12	Tension hydropneumothorax	6
16	60d	RLL	Respiratory distress	CCAM	CCAM	2	None	49
17	55d	RLL	None	CCAM	CCAM	1	None	36
18	32w	LLL	Cough	Sequestration	CCAM	0	None	3
19	39m	RLL	None	CCAM	CCAM	1	None	2
20	4w	RUL	Respiratory distress in newborn period	None	CCAM	1	None	56
21	4d	RLL	None	CCAM	CCAM	1	None	80

**Table 2:** Details of patients with histologically confirmed congenital lobar emphysema

No.	Age at surgery	Site	Preop symptom	AN diagnosis	PN diagnosis	Postop ITU days	Complications	FU (months)
1	20w	LUL	Recurrent respiratory infections	None	CLE	0	None	18
2	68w	RUL	RUL pneumonia, respiratory failure	None	CLE	1	None	32
3	2w	LUL	Respiratory distress after birth	None	CLE	3	None	21
4	20w	RUL	Recurrent respiratory infections	None	CLE	3	None	88
5	20w	LUL	Recurrent cough + dyspnoea	None	CLE	1	None	66
6	7w	LUL	Respiratory distress after birth	None	CLE	0	None	78
7	15w	RML	Recurrent respiratory infections	None	CLE	5	None	24
8	60w	RLL	None	CCAM	CCAM	0	None	2
9	8w	RUL + RML	Respiratory distress	None	CCAM	2	None	80
10	9w	LUL	Tachypnoea at 6-week	None	CLE	2	None	73
11	5w	LUL	Respiratory infection	None	CLE	7	None	62
12	10w	RML	Recurrent respiratory infections	None	CLE	3	None	17
13	20w	LUL	Recurrent respiratory infections	None	CLE	0	None	22
14	16w	LUL	Respiratory infection	None	CLE	0	None	28

**Table 3:** Details of patients with histologically confirmed intralobar pulmonary sequestrations

No.	Age at surgery	Site	Preop symptom	Antenatal diagnosis	Postnatal diagnosis	Postop ITU days	Complications	FU (months)
1	7y	RLL	Recurrent respiratory infections	None	Sequestration	2	None	54
2	48w	LLL	None	CCAM	CCAM	0	None	3
3	36w	LLL	None	CCAM	CCAM, no large vessel	1	None	38
4	9yrs	LLL	X-ray changes persisting after resolved pneumonia	None	Sequestration	0	None	11

Mean follow-up was for 39 months (range 2–88 months) and there were no complications. No patient had developed any chest deformity, although one child, who had two lobes resected, had a minor degree of chest asymmetry. All the patients were alive and well at the time of the study.

## DISCUSSION

Lung resection is an infrequent procedure in the pediatric population. Although lung resection may be performed for infective or neoplastic lesions, the vast majority of resections are now performed for CCAMs, CLEs and sequestrations. While some of these lesions may present acutely, the majority are asymptomatic[3–5] and, traditionally, such patients would only ever have presented in the event of serious complications. In recent decades however improvements in antenatal sonography have led to a huge increase in the detection of asymptomatic lesions[1,6–9] and the exact role of surgery has become highly controversial.[10,11] In the absence of comprehensive data,[10] it is difficult to assess the relative risks of operative and nonoperative management. While it is clear that many lesions are completely innocent,[12–15] it has been suggested that others may pose a significant risk of future infection[10,16–19] or even malignancy.[20–25] At this present time, surgical strategies vary widely from the “very conservative”[6] to the “very aggressive.”[1,3,4,26] In our institution, we have adopted a pragmatic practice of removing lesions that are symptomatic, large or air filled.

In recent years, video-assisted thoracoscopy (VATS) has become increasingly popular in adult thoracic practice. The first recorded use of VATS in children was by Rodgers[27] in the late 1970s and, more recently, other authors have published series showing it to be a safe and effective technique in experienced hands.[2,28,29] By and large, such lung resections have either been nonanatomical resections of small peripheral lesions or complete anatomical lobectomies,[2,29] a choice of procedure that perhaps reflects the limitations of the technique. A recent small series[30] has reported the use of VATS for lung-sparing resection, and it would appear that such a technique is at least feasible, if not exactly easy.

The potential advantages of VATS are considerable, and these include better cosmesis, less discomfort, shorter hospital stay and reduced deformity.[2,30] However, these need to be balanced against the potential disadvantages, which include greater operative risk, longer operative time, increased operative cost and unnecessary loss of normal lung tissue.[2,28] In the final analysis, open thoracotomy will always be quicker, cheaper and safer in most surgeons’ hands and the potential advantage of VATS, therefore, rest upon there being a significant incidence of the problems that it claims to avoid.

We use as small a skin incision as possible followed by a muscle-sparing approach through the 5^th^ or the 6^th^ intercostal space, depending on the location of the lesion (i.e. upper lobe or lower lobe).We routinely use a thoracic epidural for optimal pain management and always leave a chest drain postoperatively. In our series, there were no operative, perioperative or postoperative problems. The vast majority of patients made a prompt recovery and were discharged within 6 days. There has not been a single case of scapular winging or chest deformity and in only one case is there a detectable degree of chest asymmetry. This child is quite interesting in that he had both the right middle and lower lobes resected and it seems likely that his asymmetry reflects the relative absence of underlying lung tissue. With this in mind, it would seem that the best way to avoid deformity might be to do an anatomically exact lung-sparing resection, a technique which has so far proved quite difficult with VATS.

We conclude that open resection of congenital lung lesions is a simple, safe and inexpensive technique, which produces excellent results both in the short and medium term. At the present time, we feel that this should remain the “gold standard” against which newer techniques must be judged.
